# Kinesin family member 3C (KIF3C) is a novel non-small cell lung cancer (NSCLC) oncogene whose expression is modulated by microRNA-150-5p (miR-150-5p) and microRNA-186-3p (miR-186-3p)

**DOI:** 10.1080/21655979.2021.1942768

**Published:** 2021-06-30

**Authors:** Haiwang Liu, Ran Liu, Meiling Hao, Xing Zhao, Chunhui Li

**Affiliations:** a Department of Pathology, Affiliated Hospital of Chengde Medical College, Chengde, Hebei, China; b Anesthesiology Department of Southern District, Affiliated Hospital of Chengde Medical College, Chengde, Hebei, China

**Keywords:** NSCLC, miR-150-5p, miR-186-3p, KIF3C

## Abstract

This study is aimed at investigating the biological function of kinesin family member 3 C (KIF3C) in non-small cell lung cancer (NSCLC) progression and its upstream regulatory mechanism. Quantitative real-time PCR, Western blot and immunohistochemistry were adopted to examine microRNA-150-5p (miR-150-5p), microRNA-186-3p (miR-186-3p) and kinesin family member 3 C (KIF3C) expression levels. NSCLC cell proliferation, migration, and invasion were detected through cell counting kit-8 (CCK-8) assay, EdU assay, and Transwell assay. The metastasis of NSCLC cells was evaluated utilizing a pulmonary metastasis model in nude mice *in vivo*. The targeted relationship among KIF3C 3ʹUTR, miR-186-3p, and miR-150-5p were verified by dual-luciferase reporter gene assays. It was confirmed that in NSCLC tissues and cells, KIF3C expression level was increased and KIF3C overexpression promoted NSCLC cell proliferation and metastasis. Additionally, miR-150-5p and miR-186-3p directly targeted KIF3C to repress its expression. Our data suggest that KIF3C, which is negatively regulated by miR-150-5p and miR-186-3p, is an oncogenic factor in NSCLC progression.

## GRAPHICAL ABSTRACT

**Figure:**
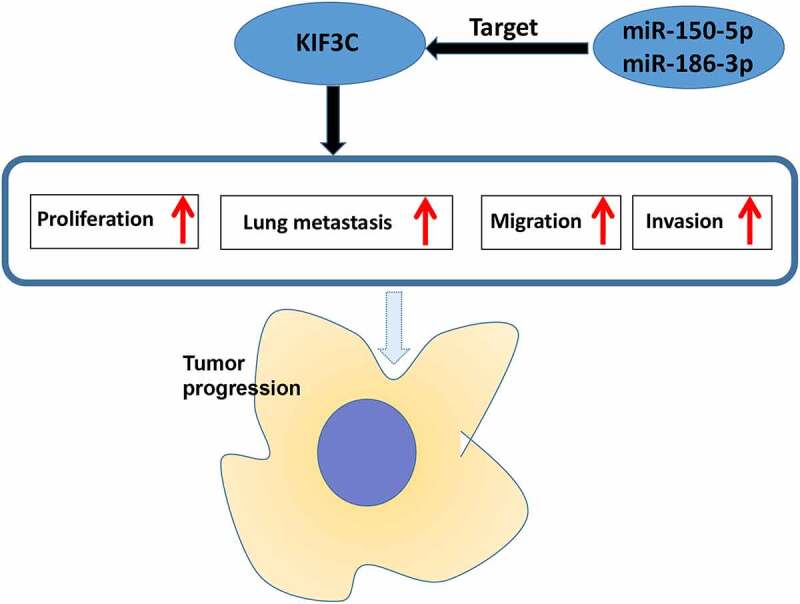

## 1. Introduction

Non-small cell lung cancer (NSCLC) cases take up about 85% of all lung cancer (LC) cases, and globally, NSCLC is the leading cause of cancer-related death in both men and women [1]. The therapies for NSCLC mainly include surgery, radiotherapy, chemotherapy, and immunotherapy [2]. Despite dramatic advances in these therapies in recent years, due to the strong metastasis ability of NSCLC cells, NSCLC patients’ five-year survival rate remains less than 15% [2]. It is urgent to delve deeper into NSCLC pathogenesis and offer a theoretical basis for exploring more effective treatment for the patients.

Kinesin family member 3 C (KIF3C) is a member of the KIF3 subfamily of kinesin superfamily proteins (KIFs), and it features prominently in regulating many biological processes [3]. KIF3 complex is associated with neuronal development and differentiation [4]. Furthermore, it regulates tumor cells’ growth, metastasis and chemoresistance [5,6]. Specifically, the up-regulation of KIF3C is accompanied by docetaxel resistance of breast cancer cells [5]. Another study shows that KIF3C expression in the low-grade glioma tissue is higher in comparison to the high-grade glioma tissue, and patients with high KIF3C expression have a longer survival time; functionally, KIF3C inhibits glioma cells’ growth through modulating the PI3K/Akt/mTOR pathway [6]. The above studies indicate that KIF3C can exert tumor-promoting effect or tumor-suppressive effect in different cancers. Through bioinformatics analysis, the current study revealed that KIF3C was up-regulated in NSCLC tissues, and it was linked to the poor prognosis of NSCLC. Thus it was hypothesized that KIF3C could probably play a cancer-promoting role in NSCLC.

Known as a type of endogenous small RNA, microRNAs (miRs, miRNAs) modulate gene expression after transcription [7,8]. They bind to the target gene’s 3ʹ untranslated region (3ʹUTR) through base pairing to repress messenger RNA (mRNA) translation or promote mRNA degradation [7,8]. MiRNAs can act as cancer suppressors or oncomiRs, and participate in the progression of multiple tumors [9–12]. Reportedly, miR-150-5p directly targets HMGA2 to repress NSCLC cell proliferation, migration, and invasion [11]. In breast cancer, miR-186-3p inhibits tamoxifen resistance and aerobic glycolysis by targeting epiregulin, thereby inhibiting breast cancer progression [12]. However, the biological functions and downstream regulatory mechanisms of miR-186-3p and miR-150-5p in NSCLC remain largely unknown.

In this study, we hypothesized that KIF3C had the potential to be the diagnostic biomarker and therapeutic target for NSCLC. We investigated the biological function of KIF3C on the malignant biological behaviors of NSCLC cells, and the regulatory function of miR-186-3p and miR-150-5p on KIF3C. Herein, we report that, KIF3C, which is negatively regulated by miR-150-5p and miR-186-3p, is an oncogenic factor in NSCLC progression.

## 2 Materials and methods

### 2.1 Ethics statement and tissue samples

From February 2018 to May 2020, normal and cancer tissue samples from 42 NSCLC patients diagnosed in the Affiliated Hospital of Chengde Medical College were collected. All patients were diagnosed with NSCLC through clinical, imaging, and pathological examinations, and they did not suffer from other malignancies. The informed consent was signed by all subjects. This study was endorsed by the Affiliated Hospital of Chengde Medical College Research Ethics Committee. Before the surgery, none of the subjects had undergone radiotherapy, chemotherapy, or targeted therapy. The tissue samples were frozen in liquid nitrogen after surgical removal and reserved for follow-up research.

### 2.2 Cell culture and transfection

Normal human bronchial epithelial (HBE) cells and NSCLC cell lines (A549, H1975, H226, and H1650 cells) were all available from American Type Culture Collection (Rockville, MD, USA). The above cells were cultured in Dulbecco’s modified Eagle’s medium (Thermo Fisher Scientific, Waltham, MA, USA) containing 100 U/ml penicillin, 0.1 mg/ml streptomycin, and 10% fetal bovine serum (FBS) (Beyotime Biotechnology Co., Ltd., Shanghai, China) in 5% CO_2_ at 37°C. The medium was refreshed every 2–3 days. When cell confluency reached 70–80%, 0.25% trypsin (Roche, Basel, Switzerland) was used for cell passage. Empty vector (pcDNA-NC), KIF3C overexpression plasmid (pcDNA-KIF3C), small interfering RNA (siRNA) targeting KIF3C (si-KIF3C), siRNA negative control (si-NC), miR-186-3p inhibitors, miR-150-5p inhibitors and their controls (inh-NC), and miR-186-3p mimics, miR-150-5p mimics and their controls (miR-NC) were constructed by GenePharma (Shanghai, China). Lipofectamine® 3000 (Invitrogen; Thermo Fisher Scientific, Inc., Waltham, MA, USA) was utilized to transfect these vectors/oligonucleotides into A549 and H226 cells. After 24 h, quantitative real-time polymerase chain reaction (qRT-PCR) was performed to detect the transfection efficiency.

### 2.3 qRT-PCR

The total RNA extraction from tissues and cells was performed using TRIzol reagent (Invitrogen; Thermo Fisher Scientific, Inc., Waltham, MA, USA), and the RNA purity and concentration were subsequently measured. The First Strand cDNA Synthesis Kit (Thermo Fisher Scientific Inc., Rockford, IL, USA) was adopted to reverse-transcribe the total RNA into cDNA. The cDNA served as a template, and the SYBR® Premix-Ex-Taq™ reagent kit (Takara, Tokyo, Japan) was applied to performed qRT-PCR on the ABI7300 system (Thermo Fisher Scientific, Waltham, MA, USA). MiRNA expression was normalized utilizing U6, and GAPDH was utilized to normalize mRNA expression. Below are the specific primers’ sequences:

KIF3C forward: 5ʹ-GGTCATGAGCAGATTCTGAC-3ʹ, reverse: 5ʹ-GAGAGCTGACCTCATTCATG-3ʹ;

miR-150-5p forward: 5ʹ-ACACTCCAGCTGGGTCTCCCAACCCTTGTACCA-3ʹ, reverse: 5ʹ-CTCAACTGGTGTCGTGGA-3ʹ;

miR-186-3p forward: 5ʹ-CGCAGGGGTTTTTTAAGTG-3ʹ, reverse: 5ʹ-CAGTTTTTTTTTTTTTTTCGGGTTTC-3ʹ;

U6 forward: 5ʹ-TGCGGGTGCTCGCTTCGGCAGC-3ʹ, reverse: 5ʹ-CCAGTGCAGGGTCCGAGGT-3ʹ;

GAPDH forward: 5ʹ-GCACCGTCAAGGCTGAGAAC-3ʹ, reverse: 5ʹ-TGGTGAAGACGCCAGTGGA-3ʹ.

### 2.4 Cell counting kit-8 (CCK-8) assay

Cells were seeded at 2 × 10^3^ cells/well into 96-well plates, and the cells were routinely cultured. Each well was added with 10 μL of CCK-8 solution (RiboBio, Guangzhou, China) at 24, 48, 72, and 96 h, respectively, followed by the incubation for 1 h. Subsequently, the absorbance at 450 nm was determined by a synergy microplate reader (BioTek, Winooski, VT, USA).

### 2.5 Transwell assays

Transwell assays were performed to detect cell migration and invasion. Cell migration assay: the cell suspension was prepared with serum-free medium. The lower chamber of Transwell system (Costar, Cambridge, MA, USA) was added with 500 μL of serum-containing culture solution, and 200 μL of cell suspension (containing about 5 × 10^4^ cells) was added to the top compartment. Then the cells were put in the incubator. After 24 h of incubation, the chambers were taken out, and the liquid in the upper chamber was removed. Cotton balls were utilized to gently wipe away the cells in the upper chamber. Next, the cells which were on the below surface of the membrane, were stained with crystal violet staining solution at room temperature for 15 min. After the cells were washed by PBS and air-dried, the cells in 5 visual fields were counted and photographed under the inverted microscope (×200). Cell invasion assay: a layer of Matrigel was added onto the membrane of the Transwell chamber in advance, and the other procedures were the same with the migration assay.

### 2.6 EdU assay

An EdU kit (Beyotime Biotechnology, Shanghai, China) was utilized to perform EdU assay following the manufacturer’s instructions. Transfected H226 and A549 cells in logarithmic growth phase were incubated in 96-well plates with 50 μmol/L EdU medium (Invitrogen; Thermo Fisher Scientific, Inc., Waltham, MA, USA). After incubation for 2 h, the cells were cleaned with PBS. Subsequently, paraformaldehyde was used to fix the cells. The addition of 200 μL of 2 mg/ml glycine and 5 min of incubation were followed by cell washing with PBS for 5 min on a shaker. Next, the cells were added with 0.5% TritonX-100 for decolorized incubation on a shaker for 10 min. Next, PBS was used to wash the cells twice, 5 min for each time. Next, the cells were stained with Apollo in darkness at room temperature for 30 min, and then stained with DAPI staining solution in darkness at room temperature for 20 min. After PBS washing, a fluorescence microscope was employed to photograph the cells.

### 2.7 Immunohistochemistry (IHC)

The NSCLC tissue samples and adjacent normal tissue samples were fixed in 10% formaldehyde and embedded in paraffin. Next, the tissues blocks were sliced, the tissues sections were deparaffinized and hydrated. The deparaffinized sections were incubated at room temperature with 1% H_2_O_2_ for 10 min to block endogenous peroxidase. After PBS washing 3 times and blocking for 1 h with immunostaining blocking solution, the sections were incubated with anti-KIF3C antibody (ab236748, 1:100) in a wet box overnight at 4°C. PBS was utilized to rinse the sections, which were then incubated with biotin-linked antiserum at room temperature for 1 h. Next, the sections were rinsed again and stained for 1 min with 3,3-diaminobenzidine hydrochloride. Ultimately, double distilled water was utilized to wash the sections, which were subsequently stained for 1 min with hematoxylin, and then the sections were observed under the microscope.

### 2.8 Dual-luciferase reporter assay

Briefly, the predicted binding sequences of KIF3C 3ʹUTR for miR-150-5p and miR-186-3p, which were predicted by bioinformatis were amplified and inserted into pmirGLO vectors (Promega, Madison, WI, USA). The wild-type (KIF3C WT) or mutant (KIF3C MUT) luciferase reporter vectors, miR-186-3p mimics, and miR-150-5mimics or the negative controls were co-transfected into 293 T cells, with Lipofectamine® 3000 (Invitrogen; Thermo Fisher Scientific, Inc., Waltham, MA, USA), respectively. After 48 h of transfection, the dual-luciferase assay system (Promega, Madison, WI, USA) was adopted for luciferase activity measurement.

### 2.9 Western blot assay

RIPA lysis buffer (Pierce, Rockford, IL, USA) was used to lyse NSCLC cells, and after high-speed centrifugation, the supernatant was collected. The BCA reagent (Pierce, Rockford, IL, USA) was applied for determining the total protein concentration, and the supernatant was heated for 10 min in a 100°C water bath to denature the proteins. Protein separation was performed by SDS-PAGE, and then the proteins were electro-transferred to the PVDF membrane (Millipore, Bedford, MA, USA). After being blocked with 5% skim milk, the membranes were incubated overnight with primary antibodies at 4°C: anti-GAPDH antibody (ab8245, 1:500, Abcam, Cambridge, UK) and anti-KIF3C antibody (ab236748, 1:500, Abcam, Cambridge, UK). After being rinsed with TBST, the membranes and the secondary antibody (goat anti-rabbit IgG coupled with HRP, ab205718, 1:1000, Abcam, Cambridge, UK) were incubated at room temperature for 1 h. After the membranes were washed with TBST again, the protein bans were developed with a hypersensitive ECL kit (Millipore, Bedford, MA, USA). An ImageJ software (NIH, Bethesda, MD, USA) was adopted to quantify the protein bands.

### 2.10 Lung metastasis model in vivo

From Hebei Medical University Animal Center (Shijizhuang, China), BALB/c nude mice (male, 4–5 weeks old) were obtained. The mice were randomly separated into two groups (KIF3C overexpression group and control group; 5 mice in each group). H226 cells (1 × 10^7^ cells/mouse) were injected via the caudal vein into each mouse. The mice were euthanized after 3 weeks, and their lungs were fixed in formalin and embedded in paraffin. Next, the tissues were sliced, and the lung tissues were stained with hematoxylin/eosin. Subsequently, the metastatic nodules in the lung tissues of the mice were observed under a microscope.

### 2.11 Statistical analysis

All experiments were conducted in triplicate. The data analysis tool was SPSS 23.0 software (SPSS Inc., Chicago, IL, USA). ‘Mean ± standard deviation’ was the expression form of the data. The differences between the two groups were evaluated by Student’s *t*-test. One-way analysis of variance was utilized to conduct the comparison among multiple groups. *P* < 0.05 signified that a difference was statistically significant.

## 3 Results

We hypothesized that KIF3C could promote the progression of NSCLC. Loss-of-function and gain-of-function models were established, and we demonstrated that KIF3C could regulate the malignant biological behaviors of NSCLC cells *in vitro* and *in vivo*. Additionally, we demonstrated that miR-150-5p and miR-186-3p directly targeted KIF3C, and the up-regulation of miR-150-5p and miR-186-3p weakened the promoting effects of KIF3C on proliferation, migration, and invasion of NSCLC cells.

### 3.1 KIF3C is significantly high-expressed in NSCLC tissues and cells

**Figure 1.**
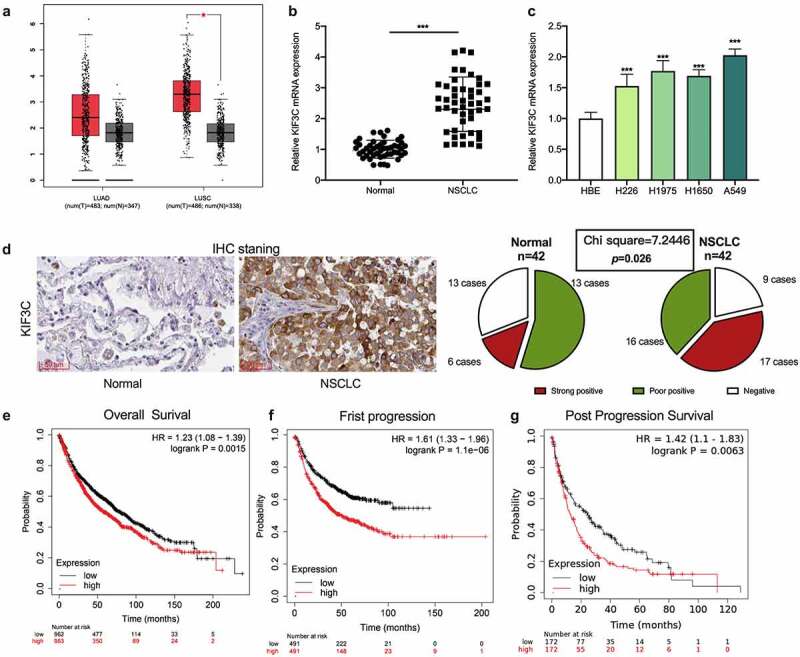
KIF3C expression is significantly up-regulated in NSCLC tissues and cells a. GEPIA database was used to analyze the expression of KIF3C in NSCLC tissues. b. Detection of KIF3C mRNA expression in NSCLC tissues and normal tissues by qRT-PCR. c. Detection of KIF3C mRNA expression in NSCLC cell lines by qRT-PCR. d. IHC method was adopted to detect KIF3C protein expression in NSCLC and adjacent tissues. E-G. KM-plotter database was searched to analyze the relationship between KIF3C expression and the overall survival time (OS), first progression (FP), and post-progression survival time (PPS) of NSCLC patients * *P* < 0.05 and *** *P* < 0.001

Firstly, GEPIA database (http://gepia.cancer-pku.cn/) was applied to analyze KIF3C expression. The data showed that KIF3C expression level in NSCLC tissues (lung adenocarcinoma: LUAD; lung squamous carcinoma: LUSC) was markedly higher compared with normal tissues (Figure 1a). Then, qRT-PCR was conducted to evaluate KIF3C expression in 42 pairs of NSCLC and para-cancerous tissues, and it was shown that KIF3C mRNA expression in NSCLC tissues was markedly up-regulated (Figure 1b). Compared with HBE, KIF3C mRNA was also significantly highly expressed in NSCLC cell lines (Figure 1c). IHC was used to further examine KIF3C protein expression in NSCLC, and it was observed that KIF3C was high-expressed in NSCLC tissues (chi-square = 7.2446, p = 0.026, Figure 1d). Through the analysis of the correlation between KIF3C expression and the patients’ clinicopathological features, it was suggested that high KIF3C expression was significantly associated with higher TNM stage (Table 1). Moreover, the KM-plotter database (https://kmplot.com/analysis/) indicated that high KIF3C expression was strongly correlated with the shorter overall survival time (OS), faster first progression (FP), and shorter post-progression survival time (PPS) of NSCLC patients (Figure 1e-g).

**Table 1.** Correlation between clinicopathological features and KIF3C expression in NSCLC

Pathological parameters	Numbers (n = 42)	KIF3C expression High (n = 21) Low (n = 21)		*p-*Value
Gender				0.4945
Male	30	14	16	
Female	12	7	5	
Age (years)				0.3167
< 50	29	16	13	
≥ 50	13	5	8	
Smoking				0.7565
Non-smoker	23	12	11	
Smoker	19	9	10	
Tumor size (cm)				0.5126
> 5	28	13	15	
< 5	14	8	6	
TNM stage				0.0278*
I + II	17	5	12	
III + IV	25	16	9	
Degree of differentiation				0.2165
Low, medium	22	13	9	
High	20	8	12	

* *P* < 0.05

### 3.2 KIF3C overexpression promotes NSCLC cell proliferation and metastasis

**Figure 2.**
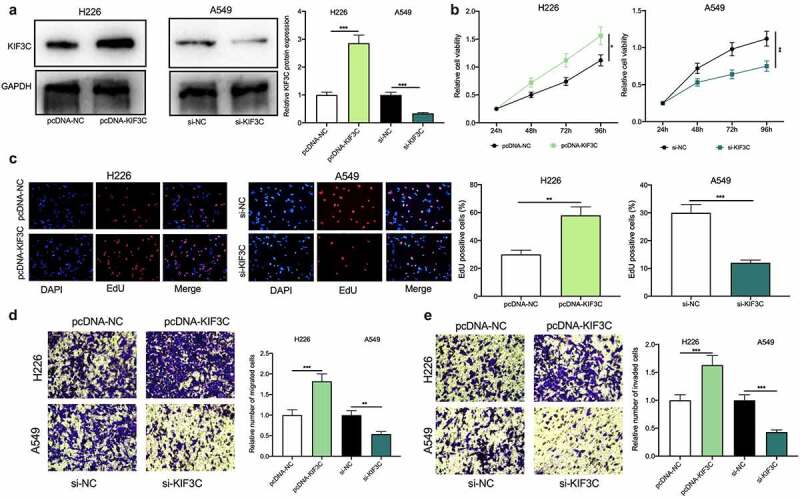
Effects of KIF3C on NSCLC cell proliferation, migration, and invasion a. H226 cells were transfected with pcDNA-NC or pcDNA-KIF3C, and A549 cells were transfected with si-NC or si-KIF3C, and KIF3C expression was detected by Western blot. B&C. CCK-8 and EdU assays were used to detect the effects of KIF3C overexpression or knockdown on cell proliferation. D&E. Transwell assays were used to detect the effects of KIF3C overexpression or knockdown on cell migration and invasion. * *P* < 0.05, ** *P* < 0.01 and *** *P* < 0.001

The above results showed that among NSCLC cells, KIF3C expression was the highest in A549 cells, whereas KIF3C expression was the lowest in H226 cells (Figure 1c). Therefore, KIF3C overexpression plasmids were transfected into H226 cells and si-KIF3C was transfected into A549 cells, and the transfection was verified by Western blotting to be successful (Figure 2a). To explore KIF3C’s biological role in regulating the malignant biological behaviors of NSCLC cells, we conducted CCK-8, EdU, and Transwell assays, and it showed that compared with the control group, KIF3C overexpression remarkably promoted cell proliferation, migration, and invasion, while KIF3C knockdown repressed cell proliferation, migration, and invasion (Figure 2b-e). Besides, the metastasis of NSCLC cells *in vivo* was evaluated using a lung metastasis model in nude mice, and it was unveiled that KIF3C overexpression facilitated the lung metastasis of H226 cells *in vivo* (Supplementary Figure 1a). The above findings suggest that KIF3C facilitates NSCLC cells’ proliferation, migration, and invasion.

### 3.3 MiR-150-5p and miR-186-3p target KIF3C

**Figure 3.**
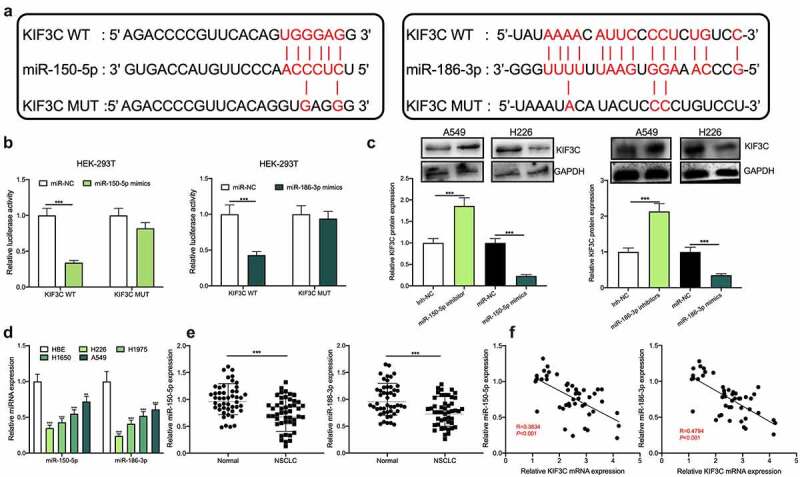
MiR-150-5p and miR-186-3p target and regulate KIF3C a. The binding sites between KIF3C 3’UTR and miR-150-5p, between KIF3C 3’UTR and miR-186-3p were predicted using the StarBase database. b. Dual-luciferase reporter gene assay was used to detect the effects of miR-150-5p and miR-186-3p on the luciferase activity of the reporters, to verify the predicted binding sites. c. Western blot was used to detect the effects of miR-150-5p and miR-186-3p on the expression of KIF3C protein. d. Detection of miR-150-5p and miR-186-3p expression in NSCLC cell lines by qRT-PCR. e. Detection of miR-150-5p and miR-186-3p expression in NSCLC and normal tissues by qRT-PCR. F. The correlation between miR-150-5p expression and KIF3C expression, and the correlation between miR-186-3p expression and KIF3C expression in NSCLC tissue samples. ** *P* < 0.01 and *** *P* < 0.001

To further explore the upstream mechanism of KIF3C, the StarBase database (starbase.sysu.edu.cn) was used to predict miRNAs that may regulate KIF3C. It was displayed that there existed binding sites between miR-150-5p (miR-186-3p) and KIF3C 3ʹUTR (Figure 3a). Subsequently, a dual-luciferase reporter gene assay was conducted for verifying the targeted relationships between KIF3C 3ʹUTR and miR-150-5p, and between KIF3C 3ʹUTR and miR-186-3p, and it was shown that miR-150-5p and miR-186-3p overexpression inhibited KIF3C WT’s luciferase activity, yet did not significantly affect KIF3C MUT’s luciferase activity (Figure 3b). Western blotting suggested that miR-150-5p and miR-186-3p overexpression markedly repressed KIF3C protein expression and the inhibition of miR-186-3p or miR-150-5p promoted KIF3C protein expression (Figure 3c). Additionally, qRT-PCR showed that miR-186-3p and miR-150-5p were dramatically under-expressed in NSCLC tissues and cell lines (Figure 3d and e). Pearson correlation analysis suggested that miR-150-5p expression and KIF3C expression in 42 cases of NSCLC tissues were negatively correlated, and miR-186-3p expression and KIF3C expression in NSCLC tissues were also negatively correlated (figure 3f).

### 3.4 MiR-150-5p targets and represses KIF3C to inhibit NSCLC progression

**Figure 4.**
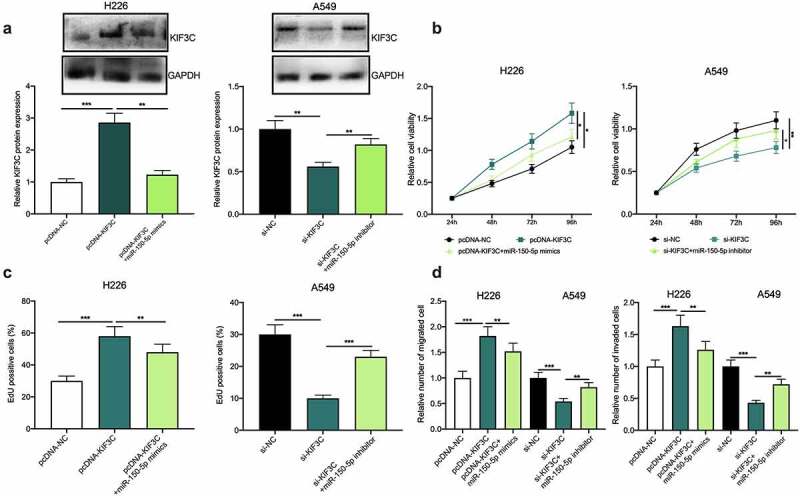
Effects of miR-150-5p / KIF3C axis on NSCLC cell proliferation, migration, and invasion. a H226 cells were co-transfected with pcDNA-KIF3C and miR-150-5p mimics, and A549 cells were co-transfected with si-KIF3C and miR-150-5p inhibitors. After transfection, KIF3C expression was detected by Western blot. B&C. CCK-8 and EdU assays were used to detect the changes in cell proliferation. D&E. Transwell assays were used to detect the changes in cell migration and invasion. * *P* < 0.05, ** *P* < 0.01 and *** *P* < 0.001

To further confirm whether miR-150-5p participated in NSCLC progression via modulating KIF3C, H226 cells were co-transfected with pcDNA-KIF3C and miR-150-5p mimics, and A549 cells were co-transfected with si-KIF3C and miR-150-5p inhibitors, and Western blotting showed that the transfection was a success (Figure 4a). Subsequently, cell proliferation, migration, and invasion were detected through CCK-8, EdU, and Transwell assays, and as shown, overexpression of KIF3C remarkably promoted H226 cell proliferation, migration, and invasion, while the transfection of miR-150-5p mimics weakened this effect (Figure 4b-d); on the other hand, knocking down KIF3C inhibited A549 cell proliferation, migration, and invasion, while the transfection of miR-150-5p inhibitors reversed this effect (Figure 4b-d).

### 3.5 MiR-186-3p targets and represses KIF3C to inhibit NSCLC progression

**Figure 5.**
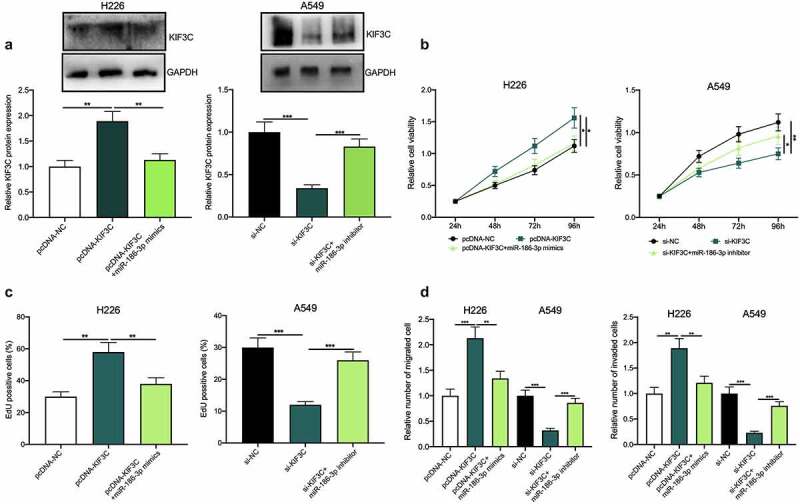
Effects of miR-186-3p / KIF3C axis on NSCLC cell proliferation, migration, and invasion a. H226 cells were co-transfected with pcDNA-KIF3C and miR-186-3p mimics, and A549 cells were co-transfected with si-KIF3C and miR-186-3p inhibitors. After transfection, KIF3C expression was detected by Western blot. B&C. CCK-8 and EdU assays were conducted to detect the changes in cell proliferation. D&E. Transwell assays were performed to detect the changes in cell migration and invasion. * *P* < 0.05, ** *P* < 0.01 and *** *P* < 0.001

Besides, to validate whether miR-186-3p was involved in NSCLC progression via targeting miR-186-3p, we co-transfected pcDNA-KIF3C+miR-186-3p mimics into H226 cells, and co-transfected si-KIF3C+miR-186-3p inhibitors into A549 cells, and the transfection was confirmed by Western blotting to be successful (Figure 5a). Next, cell proliferation, migration, and invasion were detected through CCK-8, EdU, and Transwell assays, and the functional experiments showed that KIF3C overexpression markedly promoted H226 cell proliferation, migration, and invasion, whereas the miR-186-3p overexpression weakened this effect (Figure 5b-d). Moreover, KIF3C depletion could suppress A549 cell proliferation, migration, and invasion, while the miR-186-3p inhibition reversed these effects (Figure 5b-d).

## 4 Discussion

KIF3C belongs to the kinesin superfamily protein (KIF) family. Known as a type of conserved proteins which regulate microtubule-dependent movement, KIFs feature prominently in modulating many biological processes, for instance, cell morphology, intracellular macromolecule transport, cytoskeletal dynamics, cell division, and cell migration [13–15]. Reportedly, KIF3C is highly enriched in nervous tissues such as the brain, spinal cord, and retina, and KIF3C is involved in regulating axon regeneration at growth cones after neuronal injury [16–18]. Importantly, accumulating evidence supports the regulatory function of KIF3C in cancer biology. For instance, KIF3C expression level is increased in breast cancer, and high KIF3C expression is associated with lymph node metastasis and tumor recurrence; additionally, KIF3C knockdown inhibits the EMT process via suppressing the TGF-β signal pathway and inhibits BC cell proliferation by inducing G2/M phase arrest, which suggests that KIF3C can serve as a biomarker and target for the diagnosis and therapy of breast cancer [13]. Two recently published studies report that, in glioma, up-regulation of KIF3C is associated with patients’ unfavorable prognosis; functionally, KIF3C can regulate the PI3K/AKT/mTOR signal pathway to inhibit glioma cell growth [6,19]. To our best knowledge, our study was the first to confirm that in NSCLC tissues, KIF3C was highly expressed, and its high expression was significantly associated with the patient’s adverse prognosis; furthermore, KIF3C overexpression dramatically promoted NSCLC cell proliferation, migration, and invasion, and its depletion repressed these malignant phenotypes of NSCLC cells. The findings indicate that KIF3C serves as an oncogene in NSCLC progression.

It has been reported that miRNAs play a vital role in regulating the progression of multiple cancers, and miRNAs can participate in regulating cell proliferation, differentiation, apoptosis, metabolism, inflammation, angiogenesis, and many other biological processes by modulating the expression of target genes [20]. Importantly, a lot of studies have confirmed that miRNAs serve as tumor suppressors or promoters in NSCLC [21,22]. For example, in NSCLC tissues and cells, miR-148a expression level is remarkably reduced, and low miR-148a expression is significantly related to the poor prognosis of the patients; functionally, miR-148a represses tumor cell migration and invasion via targeting Wnt1 [21]. Another study reports that miR-199 is a tumor-suppressing miRNA, and its expression is reduced in NSCLC, and miR-199 suppresses the malignant progression of NSCLC via targeting RGS17 [22]. The role of miR-150-5p in NSCLC is controversial. It is reported that miR-150-5p can repress LKB1 to promote NSCLC progression [23]. However, other studies report that miR-150-5p inhibits Wnt-β-catenin signaling via targeting GSKIP and HMGA2, or suppresses MMP14, or GLUT1 to exert its tumor-suppressive properties in NSCLC [24–26]. In the present work, our data showed that miR-150-5p was lowly expressed in NSCLC tissues, and it counteracted the oncogenic effects of KIF3C, supporting it is a tumor suppressor. Previously, the biological function of miR-186-3p in NSCLC was obscure. In cervical cancer, miR-186-3p blocks tumorigenesis via suppressing MCM2 [27]. In breast cancer, miR-186-3p overexpression markedly restrains EREG expression, thus inhibiting the drug resistance and glycolysis of cancer cells [12]. Our data suggested that miR-186-3p was down-regulated in NSCLC tissues, and its overexpression counteracted the effects of KIF3C on NSCLC cells. These results suggest that miR-186-3p is a tumor suppressor in NSCLC, which is similar with its role in cervical cancer and breast cancer [12, 27]. Importantly, this study also revealed that miR-150-5p and miR-186-3p directly targeted KIF3C and negatively regulated its expression, and our demonstrations partly explain the mechanism of KIF3C overexpression in NSCLC.

## 5. Conclusion

Collectively, our study reports that high expression of KIF3C implies unfavorable prognosis of NSCLC patients. Also, with *in vitro* and *in vivo* experiments, this study also confirms that KIF3C promotes the malignant biological behaviors of NSCLC cells. Additionally, as a downstream target of miR-186-3p and miR-150-5p, KIF3C is modulated by miR-186-3p and miR-150-5p in NSCLC cells. This study offers a novel understanding into the pathogenesis of NSCLC. However, more clinical samples are needed to further evaluate the potential of KIF3C as a prognostic biomarker for NSCLC.

## Supplementary Material

Supplemental Material

## Data Availability

The data used to support the findings of this study are available from the corresponding author upon request.

## Highlights

KIF3C expression was up-regulated in NSCLC, and it was associated with poor prognosis.

Overexpression of KIF3C promoted NSCLC cell proliferation and metastasis.

KIF3C was negatively regulated by miR-150-5p and miR-186-3p.
