# Supplementary material for: Kinesin family member 3C (KIF3C) is a novel non-small cell lung cancer (NSCLC) oncogene whose expression is modulated by microRNA-150-5p (miR-150-5p) and microRNA-186-3p (miR-186-3p)
Source: Bioengineered. 2021 Jun 30;12(1):3077–88. doi: 10.1080/21655979.2021.1942768 (PMC8806907; doi:10.1080/21655979.2021.1942768)
Supplement: Supplemental Material [file KBIE_A_1942768_SM0369.docx]

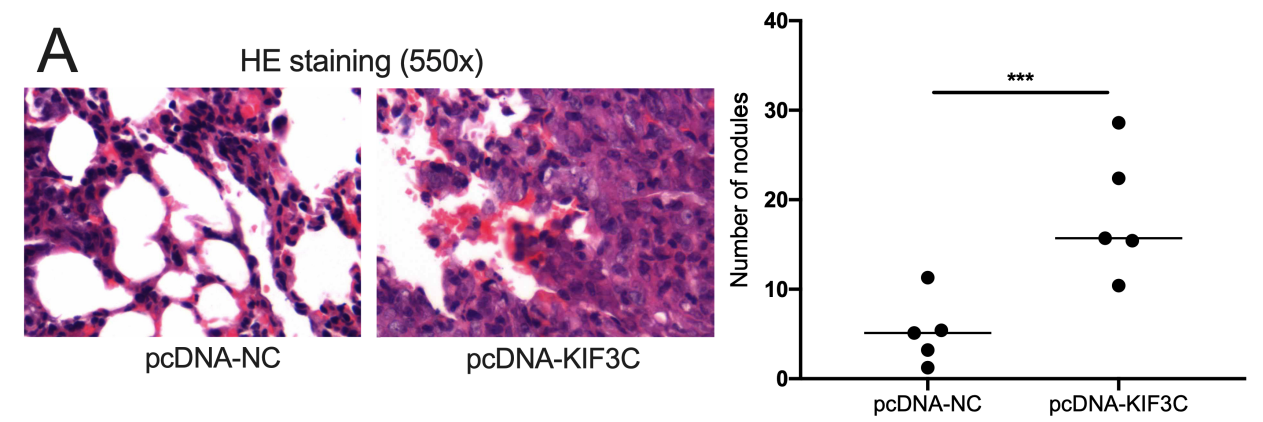


***Supplementary Figure 1***

A. Lung metastasis of NSCLC cells *in vivo* was evaluated after the nude mice were injected with H226 cells (control group or KIF3C overexpression group). The scatter diagram indicates the number of nodules per section.
